# Mechanical Properties of ZTO, ITO, and a-Si:H Multilayer Films for Flexible Thin Film Solar Cells

**DOI:** 10.3390/ma10030245

**Published:** 2017-03-01

**Authors:** Claudia Hengst, Siegfried B Menzel, Gayatri K Rane, Vladimir Smirnov, Karen Wilken, Barbara Leszczynska, Dustin Fischer, Nicole Prager

**Affiliations:** 1 IFW Dresden, SAWLab Saxony, Helmholtzstrasse 20, D-01069 Dresden, Germany; s.menzel@ifw-dresden.de (S.B.M.); g.k.rane@ifw-dresden.de (G.K.R.); 2 IEK5-Photovoltaik, Forschungszentrum Jülich GmbH, D-52425 Jülich, Germany; v.smirnov@fz-juelich.de (V.S.); k.wilken@fz-juelich.de (K.W.); 3 TU Dresden, Semiconductor and Microsystems Technology Laboratory, D-01062 Dresden, Germany; Barbara.Leszczynska@tu-dresden.de (B.L.); dustin.fischer@tu-dresden.de (D.F.); 4 Fraunhofer Institute for Electron Beam and Plasma Technology, Winterbergstrasse 28, D-01277 Dresden, Germany; Nicole.Prager@fep.fraunhofer.de

**Keywords:** silicon-based solar cells, flexible substrates, ITO, multilayers, mechanical properties

## Abstract

The behavior of bi- and trilayer coating systems for flexible a-Si:H based solar cells consisting of a barrier, an electrode, and an absorption layer is studied under mechanical load. First, the film morphology, stress, Young’s modulus, and crack onset strain (COS) were analyzed for single film coatings of various thickness on polyethylene terephthalate (PET) substrates. In order to demonstrate the role of the microstructure of a single film on the mechanical behavior of the whole multilayer coating, two sets of InSnOx (indium tin oxide, ITO) conductive coatings were prepared. Whereas a characteristic grain–subgrain structure was observed in ITO-1 films, grain growth was suppressed in ITO-2 films. ITO-1 bilayer coatings showed two-step failure under tensile load with cracks propagating along the ITO-1/a-Si:H-interface, whereas channeling cracks in comparable bi- and trilayers based on amorphous ITO-2 run through all constituent layers. A two-step failure is preferable from an application point of view, as it may lead to only a degradation of the performance instead of the ultimate failure of the device. Hence, the results demonstrate the importance of a fine-tuning of film microstructure not only for excellent electrical properties, but also for a high mechanical performance of flexible devices (e.g., a-Si:H based solar cells) during fabrication in a roll-to-roll process or under service.

## 1. Introduction

Silicon-based thin film solar cells on flexible polymer substrates such as polyethylene terephthalate (PET) are of high interest for future low-cost photovoltaic solutions ([1,2,3,4,5], and references therein), as they can be fabricated in a simple and cheap roll-to-roll (R2R) mass production process on large areas and at relatively low temperatures. In the superstrate configuration of such a solar cell, transparent barrier and front contact layers are deposited onto the flexible polymer substrate followed by a p-i-n amorphous silicon solar cell and a back contact. A permeation barrier layer is required in order to reduce aging of the solar cell due to oxygen and water vapor diffusion through the PET substrate. It has been shown that a thin layer of transparent ZnSnOx (zinc tin oxide, ZTO) is capable of reducing the water vapor transmission rate significantly while maintaining optical transparency and flexibility without cracking [6]. Typical front contact materials are thin films of transparent oxides, such as conductive InSnOx (indium tin oxide, ITO) or dielectric/metal/dielectric multilayers ([7], and references therein). Amorphous silicon and transparent oxide films are highly relevant materials not only for flexible solar cells, but also for other flexible electronics applications; for example, transistors for flexible thin-film-transistor (TFT) displays and invisible electronics on windshields and glass panels, respectively ([8] and references therein). One of the drawbacks of using transparent oxide films is that they are brittle by nature and therewith limit the flexibility of the flexible device. Uniaxial tensile [9] and buckling [10,11,12,13] tests were used to investigate the initiation of channeling cracks, buckling, and delamination of individual brittle oxide films on flexible substrates. For example, Leterrier et al. systematically examined the damage evolution of as-deposited and annealed ITO films with regard to the substrate material, the deposition gas composition, internal film stress, film thickness, and microstructure [9]. Most studies focus on the performance of single films on flexible substrates, whereas the relation between the mechanical performance of films and the failure of multilayers therefrom is of particular importance for fabrication and service purposes of a complex thin film device. Kim et al. demonstrate that the electrical resistivity of a ZTO (20 nm)/Ag (10 nm)/ITO (30 nm) multilayer electrode on a PET substrate was hardly affected by cyclic bending, whereas a 60 nm ITO reference film showed a strong resistivity increase after only a few bending cycles [7]. The authors attribute this behavior to the ductile Ag interlayer with higher failure strain. In addition, a different ITO film thickness (60 nm versus 30 nm in the multilayer stack) and a change of the ITO film morphology when deposited on Ag instead on PET may also result in a considerable change of the failure strain of the ITO film itself. Waller et al. studied the temperature influence on the failure of an ITO/a-Si:H/Ag trilayer on a polyethylennaphthalate (PEN) substrate, which is relevant for photovoltaic applications [14]. However, in their work, fracture of the weakest component of the multilayers was regarded as failing criterion for the whole coating. In contrast, in thin film solar cells, the functionality may degrade upon fracture of the absorbing layer, but is still maintained as long as the electrode layers are intact. Therefore, it makes sense to study whether the failure of the weakest component initiates cracking in all constituent layers, or whether the electrode layer remains intact as long as the applied load level is below the critical value of the electrode layer.

The first objective of the work presented here was to investigate thin film properties of individual ZTO, ITO, and hydrogenated amorphous silicon (a-Si:H) films that are relevant for their fabrication and use not only in flexible thin-film silicon solar cells, but also in other flexible electronic devices. The Young’s modulus, film morphology, and failure behavior under uniaxial tensile load were systematically studied as a function of film thickness and deposition parameters. The derived thin film properties constitute a valuable contribution to the available database of thin film parameters required for simulation and modeling of such films and multilayers therefrom. In the second part of the paper, failure mechanisms of bilayer and trilayer model systems are presented and related to the single film properties. We demonstrate that multilayer systems with altered microstructure can differ significantly in their failure behavior, which may either lead to degradation or complete failure of the solar cell. The results of this study directly contribute to the optimization of dimensioning and fabrication of compliant a-Si:H-based thin film solar cells.

## 2. Experimental Section

### 2.1. Samples

Single films of a-Si:H were prepared at a nominal temperature of T= 140 ${}^{\circ }$C by plasma enhanced chemical vapor deposition (PECVD and very high frequency PECVD) with film thicknesses ranging from 200 nm to 2000 nm. Two sets of InSnOx films (200 nm to 2000 nm) were prepared at room temperature using In${}_{2}$O${}_{3}$:SnO${}_{2}$ (95/5 wt %) targets. ITO-1 was deposited in a batch process by radio-frequency magnetron sputtering in a commercial CT II Cluster tool (VON ARDENNE GmbH) using Ar (27 sccm) and O${}_{2}$ (3 sccm, diluted 1% in Ar) as sputtering gases at a pressure of 0.12 Pa. ITO-2 was prepared by DC magnetron sputtering in a roll-to-roll (R2R) pilot plant coFlex^®^600 with a gas mixture of Ar:H (200 sccm) and O${}_{2}$ (6 sccm) at a deposition pressure of 0.4 Pa. The addition of H${}_{2}$ to the sputtering gas is known to allow for the deposition of entirely amorphous ITO films at room temperature [9]. ZnSnO${}_{x}$ films were deposited at room temperature via rf magnetron sputtering in the roll-to-roll pilot plant using a Zn52%:Sn48% target and 6 sccm O${}_{2}$ gas flow. As edge defects in the coating are known to act as crack initiation sites under tensile load, all samples were die-cut from 25 µm TEIJIN^®^TETORON^®^HB3 PET foils before deposition of the films in order to avoid film damage during sample cutting. For the deposition of the coatings, the substrates were fixed on an adhesive polymer layer that was spin-coated onto a carrier.

### 2.2. Tensile Testing

A miniaturized module for tensile and compression tests (Kammrath & Weiss, Germany) equipped with a 50 N load cell was used to obtain stress–strain curves for single films and for in-situ fragmentation tests under uniaxial tensile load. Dogbone-shaped samples with a center width of 5 mm and a long axis of 5 cm were used, with the long axis being parallel to the machine direction of the PET substrate. For tensile testing, the velocity of the traverse was 2.7 µm/s, which corresponds to a strain rate of 8.3 × 10${}^{-5}$ s${}^{-1}$. Adapted clamps were used for reduced slippage, the avoidance of film damage, and electrical contacting of the films. The elongation of the sample was measured using a non-contact laser extensometer within the center region of dogbone shaped samples where the uniaxial tensile stress was proven to be homogeneous using finite element simulations (Supplementary Figure S1). The samples were prestrained before measurement with a force 0.1 N ≤F≤ 0.2 N. In order to obtain the stress–strain curves of single films, we followed the approach described in [15,16], assuming that the tensile stress is homogeneous across the cross-section of the film within the center region of the sample. The stress in the film is obtained by subtracting the contribution of the substrate $F_{s}$ from the total force $F_{total}$:

(1) $$\sigma_{f}=\frac{1}{wt_{f}}[F_{total}(\epsilon_{i})-F_{s}(\epsilon_{i})],$$

for each strain value $\epsilon_{i}$, with *w* being the sample width and $t_{f}$ the film thickness. In order to determine $F_{s}\left(\epsilon_{i}\right)$, the force–strain curves of reference substrates are obtained after removal of the film by wet-chemical etching. This ensures that the reference substrates experienced the same temperature load and ion bombardment during film deposition as the according film–substrate composites. Note that by using Equation (1) for calculating the film stress, values are only reliable for strains smaller than the crack onset strain (COS) of the film. The Young’s moduli of the films were determined by measuring the slope of the stress–strain curves for strains 0.0005 ≤ϵ≤ 0.0025 and ϵ≤COS. As the properties of elastic substrates may vary significantly, at least two samples were used to determine an averaged reference stress–strain curve. For a minimum statistic validation of the thin film parameters, 2–3 thin film samples were measured when available. The critical strain at which cracks initiate was determined via in-situ tensile tests at a digital Keyence microscope in transmission mode for non-conductive a-Si:H and ZTO films. The crack onset of the conductive ITO was detected in-situ by electrical resistance measurements, with the clamps being used as electrical contacts. A failure criterion of ≈10% change of electrical resistance was chosen and verified optically.

## 3. Results and Discussion

### 3.1. Film Characterization

**Figure 1:**
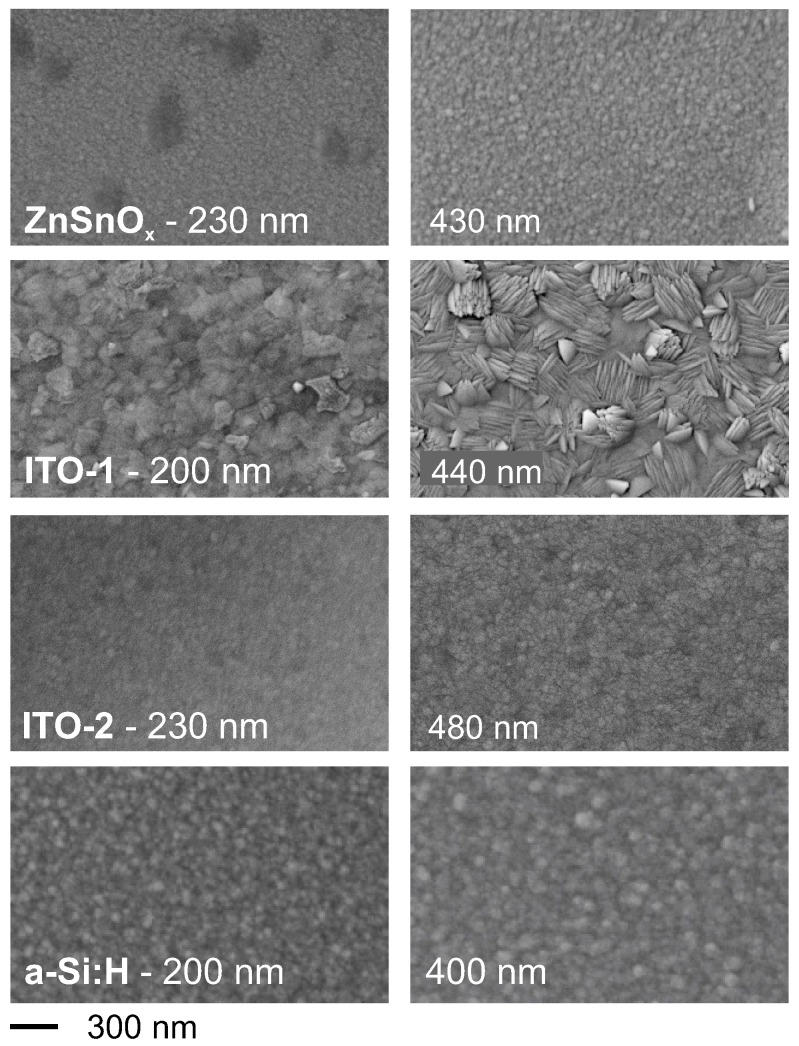
SEM images of zinc tin oxide (ZTO), indium tin oxide (ITO), and a-Si:H films for film thicknesses of 200 nm and 400 nm.

The scanning electron microscopy images shown in Figure 1 compare the film morphology of the different coatings for selected film thicknesses of approximately 200 and 400 nm.

**Figure 2:**
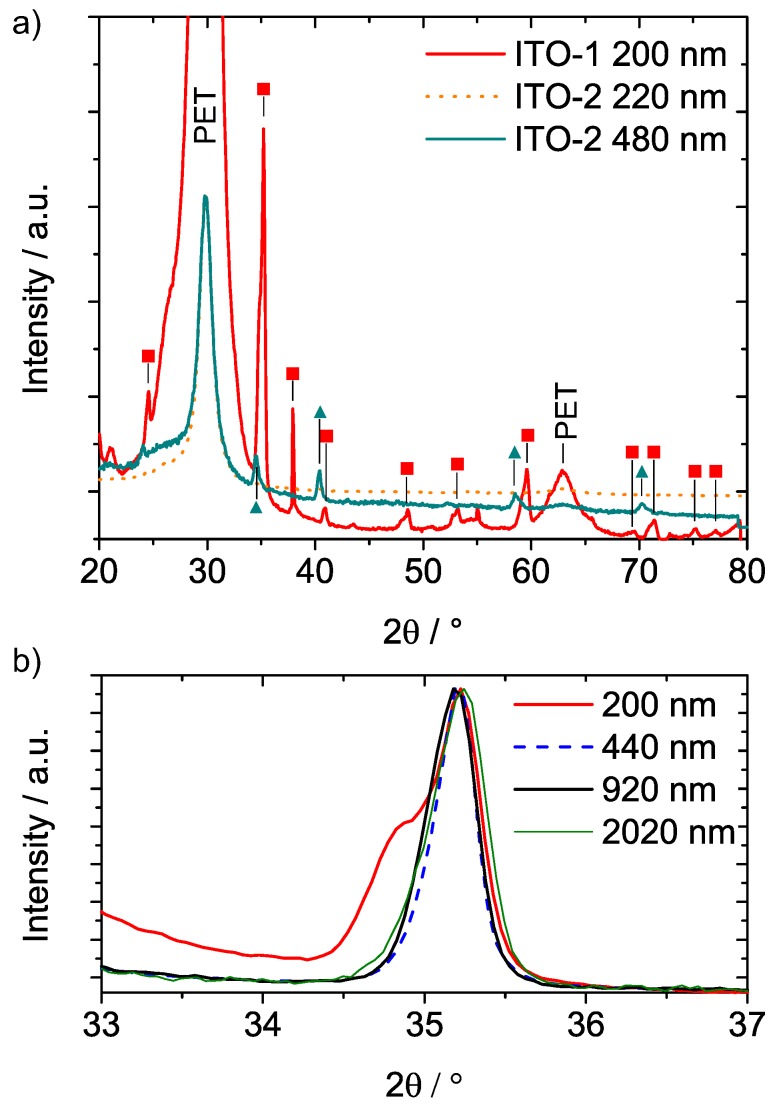
(**a**) X-ray diffraction patterns of representative ITO-1 and ITO-2 coatings. The peaks are assigned to the In${}_{1.9}$Sn${}_{0.05}$O${}_{2.95}$ phase by filled squares and to the Sn(Sn${}_{2}$In${}_{4}$)O${}_{12}$ phase by filled triangles; (**b**) (222)-diffraction peak of ITO-1 films with varying thickness. For a better comparison of the full width at half maximum (FWHM), the peaks are normalized to the maximum intensity of the (222) peak. For the 200 nm film, the shoulder of the peak belongs to the Sn(Sn${}_{2}$In${}_{4}$)O${}_{12}$ phase.

Except the ITO-1 coatings, all the other films are rather smooth without a visible grain structure. In the case of ITO-1, grains are weakly visible in the 200 nm film. The 400 nm ITO-1 coatings exhibit the characteristic grain–subgrain structure, which is typically observed in sputter-deposited ITO films due to the crystalline-plane-dependent resputtering of ITO films during deposition [17]. Crystallinity was verified for all ITO-1 films (Figure 2) by X-ray diffraction using a Co-K${}_{\alpha }$ radiation source.

**Figure 3:**
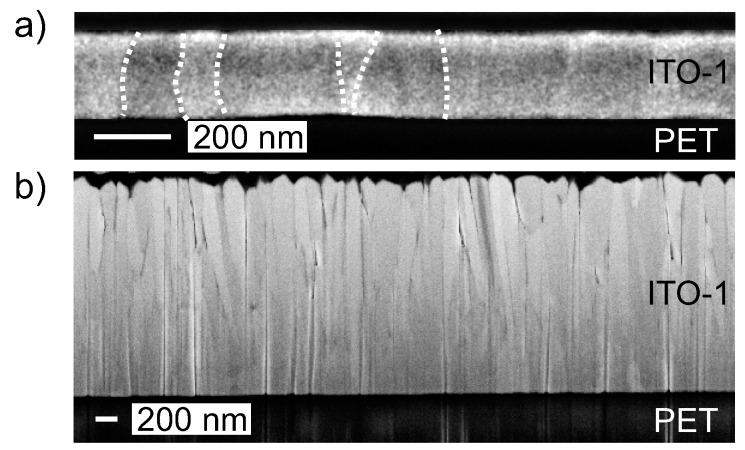
Focused ion beam (FIB) cuts of (**a**) ≈200 nm and (**b**) ≈2020 nm ITO-1 films. For better visualization, the contrast was enhanced in (a) and grain boundaries are indicated by dotted lines in the left image section. PET: polyethylene terephthalate.

As Figure 2a exemplarily shows for the 200 nm film, all the ITO-1 diffraction spectra show peaks belonging to the In${}_{1.9}$Sn${}_{0.05}$O${}_{2.95}$ phase. Additionally, for a film thickness of 200 nm, small side peaks in the diffraction spectrum of ITO-1 indicate the presence of a small fraction of a second Sn-rich Sn(Sn${}_{2}$In${}_{4}$)O${}_{12}$ phase. A slight but systematic increase of the full width at half maximum (FWHM) is observed (Figure 2b) when increasing the thickness from 440 nm to 2020 nm, which is due to an increase of the defect density [18]. A decrease of the grain size parallel to the film normal could also explain the broadening of the FWHM. However, focused ion beam (FIB) cuts through 200 and 2020 nm films (Figure 3) prove that grains extend over the whole film thickness in all cases.

In the case of ITO-2, no significant morphological changes were observed when increasing the film thickness from 200 nm to 400 nm. The addition of hydrogen to the sputtering atmosphere widely suppressed crystallization in the 220 nm film. The XRD pattern of the 480 nm film showed small diffraction peaks belonging to the Sn(Sn${}_{2}$In${}_{4}$)O${}_{12}$ phase. This indicates an amorphous film containing some small Sn(Sn${}_{2}$In${}_{4}$)O${}_{12}$ crystallites.

**Figure 4:**
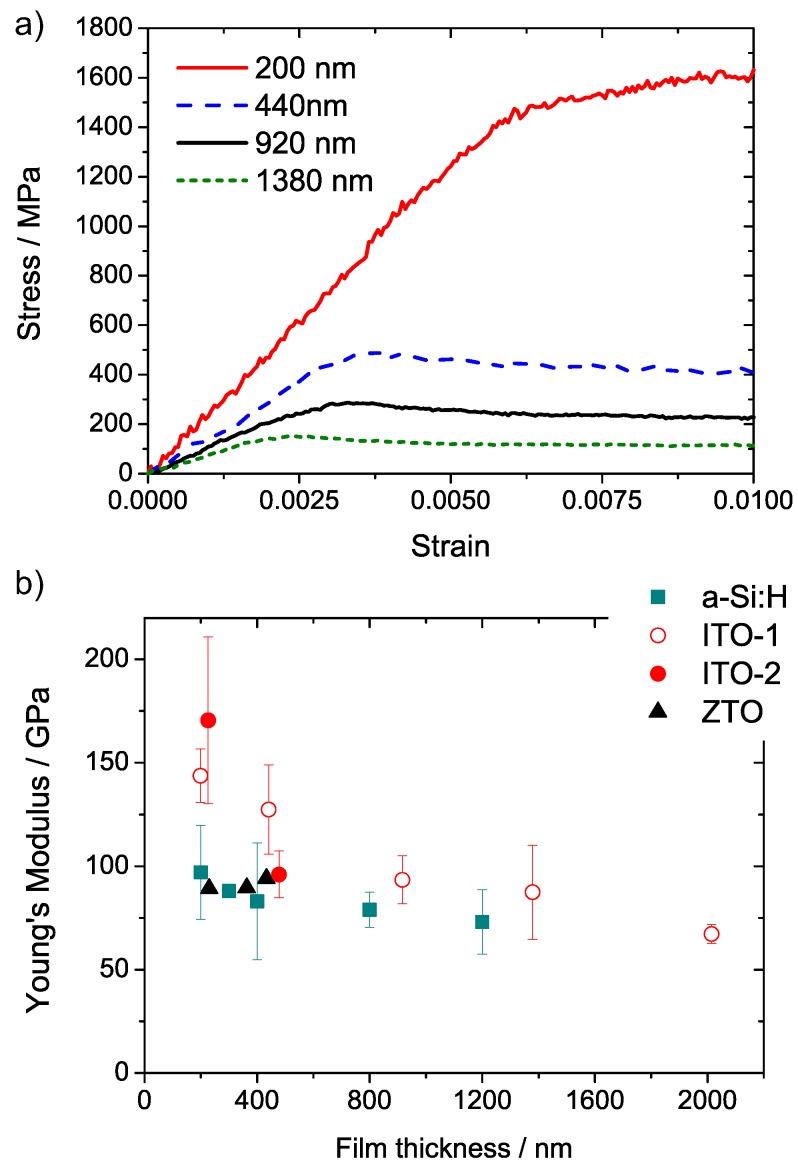
(**a**) Characteristic stress–strain curves for ITO-1 films of varying thickness after subtracting the substrate contribution. For comparison, the initial strain due to prestraining was shifted to zero. Stresses beyond the linear region at small strains do not correspond to the applied stress in the coating due to film rupture; (**b**) Young’s moduli that were extracted from the initial slope of stress–strain curves. Error bars indicate the standard deviation for averaged values in the case of several measurements.

Figure 4a shows stress–strain curves for ITO-1 films of varying thickness. Up to the crack onset strain, the tensile stress supported by the film increases linearly with increasing strain. For strain values larger than the COS, the stress values no longer represent the actual stress in the film, as Equation (1) is no longer valid. The decreasing slope in the linear-elastic regime with increasing thickness demonstrates the strong thickness-dependence of the Young’s modulus of the ITO coatings. The measured Young’s moduli of the different coatings are summarized in Figure 4b.

It is apparent that ITO exhibits the strongest variation of the Young’s modulus with coating thickness. Surprisingly, the Young’s modulus is decreasing as the film thickness is increasing. This can be explained by an increased defect density in films of larger film thickness, as shown above. The amorphous ITO-2 film shows a slightly higher Young’s modulus for 200 nm compared to the crystalline ITO-1 coating. An increase of the Young’s modulus of films sputtered under hydrogen atmosphere was also reported by Zeng et al., attributed to an increased porosity [19]. The decrease of Young’s modulus with increasing thickness of the ITO-2 from 200 nm to 400 nm might be due to the presence of small crystallites in an amorphous matrix (Figure 2a). A verification of this trend for larger film thicknesses was not possible due to the failure of thicker films as a consequence of thermo-mechanical stress during the R2R deposition. The Young’s moduli of a-Si:H films are in the range of 80 ± 20 GPa which was previously reported for amorphous silicon films [20]. For film thicknesses below 400 nm the Young’s modulus slightly decreases with increasing thickness. Kuschnereit et al. showed that the Young’s moduli of a-Si:H films measured via broadband surface acoustic wave spectroscopy varied between 14 GPa and 134 GPa, depending on the hydrogen content [21]. A difference in hydrogen content might explain the observed thickness dependence of the Young’s modulus. On the other hand, Pantchev et al. showed that the amount of bonded and mobile molecular hydrogen does not vary significantly with the film thickness [22]. Waller, et al. reported a Young’s modulus of 125 GPa for a 230 nm a-Si:H film [14]. The Young’s moduli of the ZTO coatings are in the order of 90 GPa, and reveal no pronounced change within the studied range of film thicknesses.

### 3.2. Failure of Single Films

**Figure 5:**
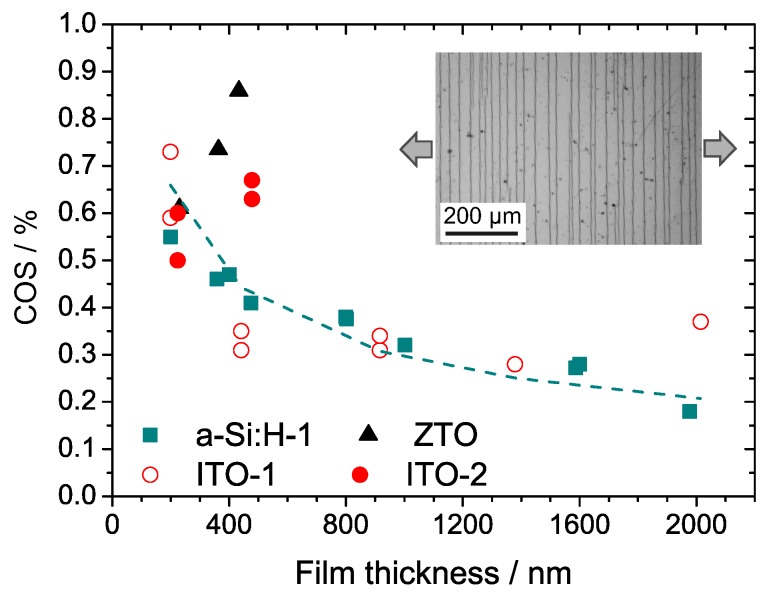
Crack onset strain (COS) for single films of a-Si:H, ITO-1, ITO-2, and ZTO with varying film thickness on 25 µm PET substrates. The dashed line indicates the proportionality of the critical strain and $1/\sqrt{t_{f}}$ for a-Si:H coatings. The inset shows an example of channeling cracks in a ca. 400 nm ITO-1 film. Arrows indicate the direction of the applied tensile load.

In-situ tensile experiments revealed that channeling cracks were initiated at defect sites as well as at the sample edges. A slight increase of the applied strain after crack initiation resulted in a fast crack propagation perpendicular to the tensile stress. This behavior is typical for the failure of brittle films on flexible substrates ([10] and references therein). In general, cracks initiate at flaws located at the film surface or within the interior of the film. According to the weakest link model, the crack onset strain is expected to decrease for increasing film thickness [23]. A decrease of the COS was observed for a-Si:H and ITO-1 coatings (Figure 5).

For amorphous a-Si:H-films, the thickness dependence of the crack onset strain approximately follows

(2) $$\epsilon_{c}\approx \frac{1}{t_{f}},$$

with $t_{f}$ being the film thickness [23]. The agreement between the simplified shear lag model and experimental data implies that the fracture toughness of the a-Si:H films is independent of the film thickness. In the case of ITO-1, Equation (2) alone fails to describe the change in COS when increasing the film thickness. A strong decrease of COS is observed for increasing film thicknesses up to 400 nm. For ITO-1 films with larger thickness, the COS was observed to be nearly constant. This implies that the failure of the ITO-1 coatings is rather dominated by microstructural changes and deposition induced stress than by the presence of defects. Note that all films studied here are macroscopically under compressive stress, which is mainly due to the different thermal expansion behavior of the film and the PET substrate. Surprisingly, the ZTO and ITO-2 coatings deposited in a R2R process show the opposite trend: the crack onset strain increases as the film thickness is increased. This might be due to an increase of compressive stress for longer deposition times in films that were fabricated using the R2R process. A higher film thickness requires longer deposition times that in turn result in a stronger heating of the substrate due to ion bombardment and insufficient cooling. Additionally, a tensile preload required for guiding the tape acts on the substrate. After cooling and release of the preload, large compressive stresses build up in the film.

### 3.3. Failure of Multilayer Films

**Figure 6:**
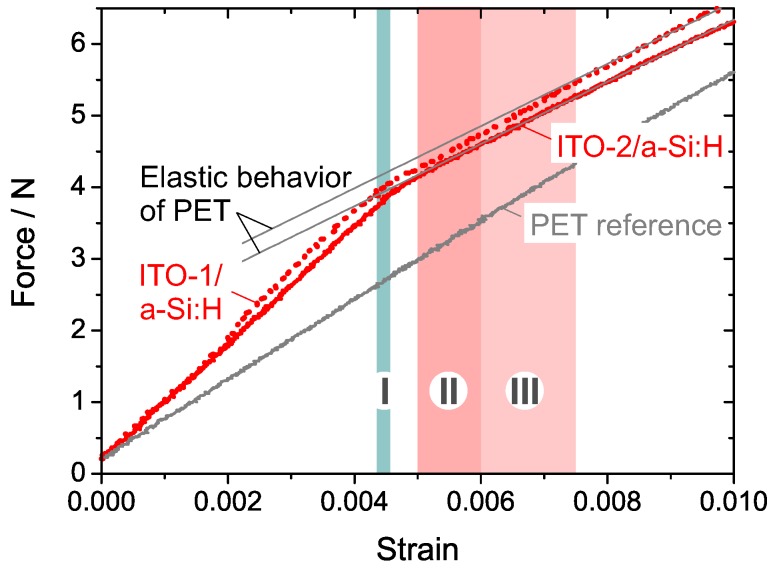
Force–strain curves of two bilayer systems containing ITO-1 (dotted line) and ITO-2 (solid line) and of a PET (HB3) reference substrate (grey solid line). Color bars indicated the range of measured COS values for single films of I: 400 nm a-Si:H, II: 200 nm ITO-2, and III: 200 nm ITO-1. To allow for a better comparison, lines with the slope of the reference curve are superimposed to the bilayer curves.

At first, we consider the failure of ITO (≈200 nm)/a-Si:H (≈400 nm) bilayer systems under uniaxial tensile load. Under increasing tensile load, two cases of failure are thinkable: (i) the constituent layer holding the lowest COS value cracks first, followed by the failure of the second layer at larger strain. In this case, the 400 nm a-Si:H layer is expected to fail first as it has a lower COS compared to the values of 200 nm ITO-1 or ITO-2; (ii) The bilayer stack fails as a whole at a strain value which is either comparable to the COS of the weakest layer or at a value which is critical for the bilayer. Figure 6 compares the force–strain curves of bilayers containing ITO-1 and ITO-2 films and of PET as a reference substrate.

The force–strain curve of the ITO-2/a-Si:H is linear until it reveals a kink at ϵ≈0.46% which is in the order of the COS of a 400 nm a-Si:H film. For higher strains, the slope of the linear curve matches that of the PET reference curve, which indicates that the deformation is fully realized by straining the elastic substrate, whereas the bilayer stack has failed. In contrast, the ITO-1/a-Si:H curve starts to deviate from the ITO-2/a-Si:H curve at ϵ≈0.2%. The origin of this deviation is still unclear, but was proven to be reproducible. At ϵ≈0.44%, the ITO-1 curve possesses a second kink, but the slope of the force-strain-curve is still higher compared to the elastic substrate. This indicates that parts of the bilayer stack still contribute to the elastic deformation. For ϵ≥0.73%, the slope finally matches that of the PET substrate, indicating that all the layers have cracked. Note that the position of the second and third kink in the force–strain curve of the ITO-1/a-Si:H bilayer stack match the range of the crack onset strains for single a-Si:H and ITO-1 films, respectively. For this reason, it can be assumed that for the ITO-1/a-Si:H system, the a-Si:H layer—being the weakest component of the multilayer—fails first, while the ITO-1 film is still intact.

**Figure 7:**
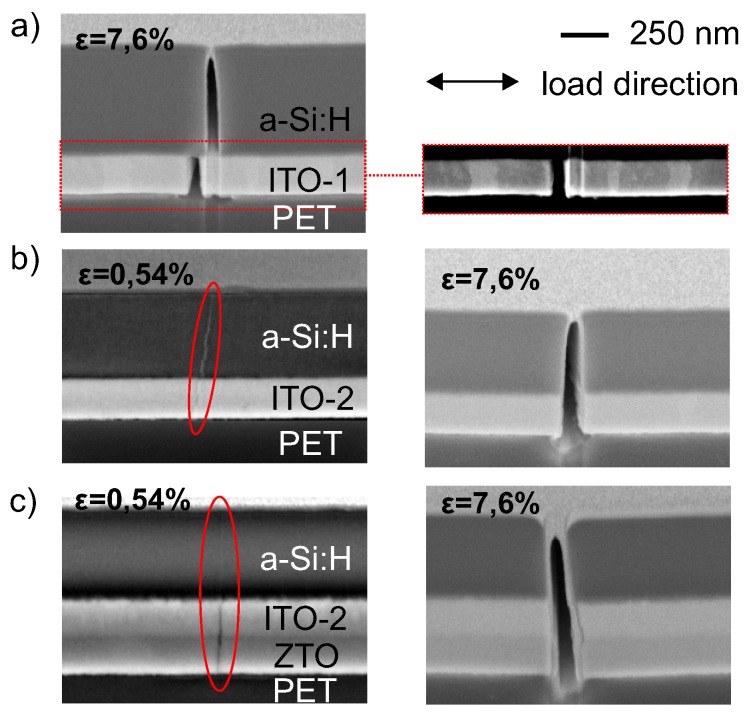
Cross-sections of bilayers of a-Si:H and (**a**) ITO-1 or (**b**) ITO-2 films on PET after tensile loading perpendicular to the crack path up to a maximum strain value as indicated in the images. The right image in (a) shows a detail of the layer stacks with enhanced contrast for a better visualization of grains; (**c**) A crack across a ZTO/ITO/a-Si:H trilayer after maximum applied strain as indicated in the images.

In order to verify the deduced failure behavior for the two bilayer systems, cross-sections of strained bilayer–substrate composites were prepared using a focused ion beam. Figure 7a,b compare the cross-sectional views of bilayers from the two sample sets. Assuming a homogenous stress state across a multilayer stack, a crack can be expected to be initiated in the weakest component. With the stress being applied in the film plane and with the material being brittle, this crack runs through the film thickness until it reaches an interface. Whether a crack runs straight through the interface and propagates in the ITO-1 film or whether it gets deflected along the interface now depends on the elastic properties of both films, the film toughness of ITO-1, and the toughness of the a-Si:H/ITO-1-interface. A quantitative determination of the film and interface toughness is beyond the scope of this study. In the following, a phenomenological interpretation of the crack evolution is given according to the cross-sectional images and the measured COS (Figure 5).

In ITO-1/a-Si:H coatings, a crack is initiated in the top a-Si:H layer, as it shows the lowest COS. An immediate propagation into the ITO-1 is unlikely, as this film can support larger strain until failure. At the interface of a-Si:H and ITO-1, the crack gets deflected and continues to propagate along the interface until it again gets deflected into the ITO-1 film. Although grain boundaries are not clearly visible in the SEM image, it is assumed that a crack travels along interface until it reaches the nearest grain boundary of the ITO film. This argument is supported by an average lateral offset between the crack path in the a-Si:H and underlying ITO film of approximately 90 nm while the grain size was on the order of 100–250 nm. The crack pattern of the ITO-1/a-Si:H bilayer therewith supports the assumption drawn from Figure 6 that both layers fail separately at strain values which are comparable to that of the single films. In contrast, cracks run straight across the whole ITO-2/a-Si:H coating. This is consistent with the observation of only one kink in the corresponding force–strain curve discussed above. However, it cannot be clearly distinguished whether the crack propagation straight across the film stack is due to a different interface toughness of the ITO-2/a-Si:H interface or due to the fact that the COS value of 200 nm ITO-2 is similar to that of 400 nm a-Si:H. Figure 7c shows the crack path for a trilayer system of ZTO (200 nm)/ITO-2 (200 nm)/a-Si:H (400 nm). Again, the crack runs straight through the whole trilayer stack. Failure occurs at ϵ≈0.41 % (stress–strain curve not shown). Hence, the failure mode of the trilayer was found to be very close to that of the ITO-2/a-Si:H bilayer.

## 4. Conclusions

The elastic behavior of single ITO, a-Si:H, and ZTO films of various film thickness on flexible low-cost PET substrates were studied and related qualitatively to the film morphology—particularly to the crystallinity, the phase, and the size and shape of the grains. Two sets of ITO films—a crystalline ITO-1 series and amorphous ITO-2 films—were prepared by a batch and an R2R sputtering process, respectively. The Young’s modulus of both ITO-films was found to decrease with increasing thickness, whereas a-Si:H films and ZTO films show a nearly constant Young’s modulus within the investigated thickness range. The films were found to crack via the formation and propagation of channeling cracks perpendicular to the tensile load direction. Thereby, the crack onset strain of a-Si:H decreased with increasing film thickness, therewith increasing the number of defects that initiate crack formation. Interestingly, the COS of films prepared by R2R sputtering increased with increasing film thickness, which was attributed to increasing compressive stress in the films. Finally, the failure behavior of ITO/a-Si:H bilayers was studied depending on the ITO film morphology. Whereas the individual layers of crystalline ITO-1/a-Si:H films failed separately from each other at strain values that are comparable to the single film COS, the amorphous ITO-2/a-Si:H stack cracks as a whole at strains comparable to the COS of the weakest link. A ZTO/ITO-2/a-Si:H trilayer was found to behave similarly to the corresponding bilayer. From an application point of view (e.g., for flexible a-Si:H based thin film solar cells), the failure behavior of the ITO-1/a-Si:H system is preferable, as the electrical contact is not lost as long as the tensile strain is smaller than the value of the crack onset strain of the electrode layer. Additionally, the adhesion between ITO-1 and a-Si:H was found to be (qualitatively) stronger compared to that of ITO-2 and a-Si:H.

## Supplementary Materials

The following are available online at www.mdpi.com/1996-1944/10/3/245/s1.
