# Supplementary material for: Mechanical Properties of ZTO, ITO, and a-Si:H Multilayer Films for Flexible Thin Film Solar Cells
Source: Materials (Basel). 2017 Mar 1;10(3):245. doi: 10.3390/ma10030245 (PMC5503339; doi:10.3390/ma10030245)
Supplement: Supplementary file 1 [file materials-10-00245-s001.pdf]

Supplementary Materials: Mechanical Properties of ZTO, ITO, and a-Si:H Multilayer Films for Flexible Thin Film Solar Cells

Claudia Hengst, Siegfried B Menzel, Gayatri K Rane, Vladimir Smirnov, KarenWilken, Barbara Leszynska, Dustin Fischer and Nicole Prager

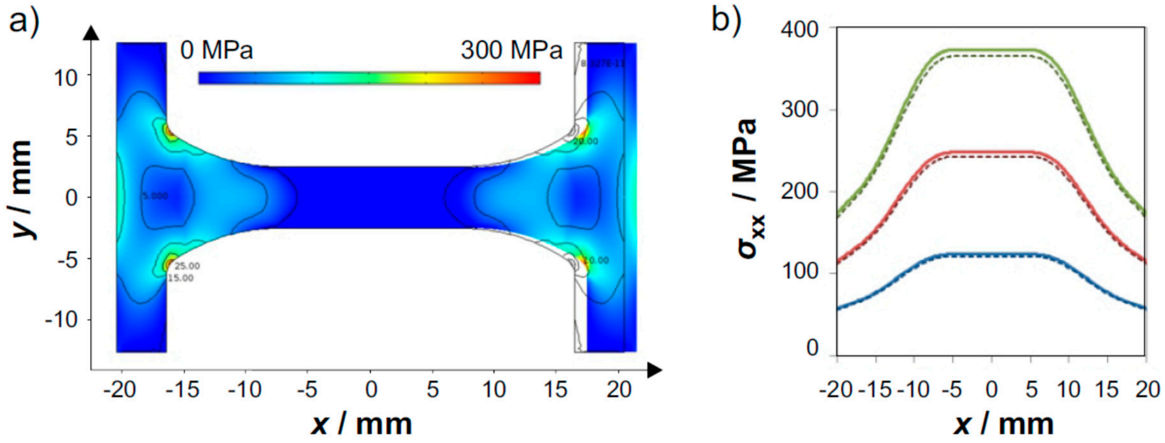

Figure S1. (**a**) Distribution of the y-component of the simulated von Mises stress in a 800 nm a-Si:H film on a 25 μm PET substrate. The sample was strained along *x* with a total elongation of Δ*L* = 1000 μm. The sample shape is comparable to the dogbone shape used in tensile experiments; (**b**) Comparison of the x-component of the von Mises film stress for *y* = 0 and different elongations as indicated in the graph. Solid lines represent the case that the Poisson number of the film and the PET substrate are equal $\nu_f = \nu_s = 0.33$. Dashed lines correspond to the case that $\nu_f = 0.22$ and $\nu_s = 0.33$. It can be seen that within a center region of ≈ 14mm the stress in the film is homogeneous.
